# Interactions between risky decisions, impulsiveness and smoking in young tattooed women

**DOI:** 10.1186/1471-244X-13-278

**Published:** 2013-11-01

**Authors:** Semion Kertzman, Alex Kagan, Michael Vainder, Rina Lapidus, Abraham Weizman

**Affiliations:** 1 Forensic Psychiatry Division, Beer-Ya’akov-Ness Ziona Mental Health Center, Beer Yaakov, Israel; 2 Sackler Faculty of Medicine, Tel Aviv University, Tel Aviv, Israel; 3 Hermeneutics and Culture Department, Psychoanalysis and Interpretation program, Bar-Ilan University, Ramat Gan, Israel; 4 Generation5, Toronto, Canada; 5 Comparative Literature Department, Bar-Ilan University, Ramat Gan, Israel; 6 Research Unit, Geha Mental Health Center, Petach Tikva, Israel; 7 Felsenstein Medical Research Center, Tel Aviv, Israel

**Keywords:** Tattoo, The Iowa Gambling Task, Impulsivity, Smoking

## Abstract

**Background:**

According to previous studies, one of the common problems of everyday life of persons with tattoos is risky behavior. However, direct examination of the decision making process, as well as factors which determine women’s risk-taking decisions to get tattoos, have not been conducted. This study investigates whether risk taking decision-making is associated with the self-assessment impulsiveness in tattooed women.

**Methods:**

Young women (aged 18–35 years) with (N = 60) and without (N = 60) tattoos, performed the Iowa Gambling Task (IGT), as a measure of decision-making processes, as well as completing the Barratt Impulsivity Scale (BIS-11).

**Results:**

Tattooed women showed significantly higher scores in the BIS-11 and preference for disadvantageous decks on the IGT compared to non-tattooed women. There was no significant correlation between risky decision-making in the IGT and BIS-11 impulsivity measures. A significantly higher rate of smoking was observed in the tattooed women. However, the analysis did not reveal a group effect after adjustment for smoking in the IGT and the BIS-11 measures.

**Conclusions:**

The present study was specifically designed to resolve questions regarding associations between impulsiveness and risky decision-making in tattooed women. It shows that in tattooed women, risky decisions are not a direct result of their self-reported impulsiveness. Smoking does not explain the psychometric differences between tattooed women and controls.

## Background

Tattooing is s phenomenon becoming increasingly common among individuals [1]. Although tattooing is often suggested to be a masculine trait [2,3] it has been reported that women make up to 45-65% of the tattooed population [4,5]. Though the popularity of body modification increases, psychosocial data about tattooing behavior are few and controversial [6,7].

Why do some women get tattoos despite possible negative consequences?

It was suggested by some physicians that finding a tattoo during physical examination should alert to the possibility of an underlying wide range of psychopathological conditions [8-10]. Tattoos are associated with personality characteristics [11] such “sensation seeking” and impulsiveness [12-15] and with cluster B personality disorders [16,17]. Tattooed subjects rated themselves as more adventurous, creative, artistic, individualistic, attractive and risk-takers than those without tattoos [15,18,19]. Participants who experienced sexual abuse often stated that they use tattoos to overcome certain traumatic experiences, and those with high numbers of tattoos were characterized by an addiction-like drive to continue body modification [6].

Tattooing was also found to be associated with a wide range of impulsivity-related behaviors such as: violence, weekly alcohol consumption, illicit drug use, dropping out of school, greater numbers of lifetime sexual partners, unprotected sex, suicidal attempts, death by homicide [20-28] and shoplifting [15]. According to previous studies, one of the common problems of everyday life of persons with tattoos is risk-taking behavior [12,29,30] leading to medical complications (e.g., potential diseases, allergies, or infections after tattooing; [31], especially the transmission of hepatitis C [32]. High tattoo prevalence was found among unemployed young women who do not live in a stable partnership [33].

However, direct examinations of the decision-making process, as well as factors that determine the risk-taking decision to get tattoos, have not been conducted amongst women.

Decision-making is a cognitive function concerned with the process of reflecting on the consequences of a certain choice [34]. Despite evidence of a range of behavioral deficits as the precursors of getting tattooed, the basis for the risk-taking behavior has not been completely characterized. The basis of decision strategy in tattooed subjects was not previously investigated.

Most existing theories suggest impulsiveness as the main reason for getting a tattoo. It seems plausible to assume that impaired decision making reflects a variety of impulse control problems [35]. However, the association between the decision-making process and impulsivity is unclear [36,37] and their role in tattooed populations has not been investigated.

The Iowa Gambling Test (IGT) is designed as a measure of risky decision making [38]. This task is the most popular measure of decision-making processes (for a review, see [39]) that mimics real-life decisions [40]. In IGT, each choice is ambiguous, at least initially, with regard to the outcome. Effective performance on the IGT depends on the ability to learn to avoid risky card decks and instead develop a preference for safe ones [41,42].

Risky decision-making may be a predictor of tattooing behavior, because both result from interacting impulsiveness traits. Some authors found an association between self-reported impulsiveness and decision making performance [36,43-46]. However, no such association was found by others between impulsivity and risky decision-making [47-53]. If indeed impulsivity and risky decision-making decompose into two independent constructs - then each can predict tattooing behavior.

Previous studies showed that tattooing behavior was associated with higher smoking rates as compared to the general population [15,54]. A large body of research has examined the association of cigarette use with individual differences in the decision making processes and impulsivity. A review of this literature suggests that both risky decision-making [55] and impulsiveness [56] constitute important correlates of tobacco use. Tobacco use can be a significant confounder that mediates among risky decision-making, impulsiveness and tattooing behavior. Such association between tattooing behavior and smoking may be largely related to non-rational decision making and non-planning behavior. The present study aimed to evaluate the association of risky decision-making and impulsiveness with tobacco use in tattooed women. We hypothesized that measures of risky decisions and impulsiveness would be associated with tattooing behavior, but that these associations are independent of tobacco use.

The major issues of the present study were: (1) Do personality trait such as impulsiveness and risky decision-making interact in predicting tattooing behavior? (2) Which of them is the best predictor for tattooing behavior?

To this end, we compared the level of impulsiveness as measured by the Barratt Impulsivity Scale (BIS-11) and by the IGT, in healthy tattooed women with that of healthy untattooed women. We predicted that: (1) women with tattoos would report higher impulsiveness scores on the BIS-11 than women without tattoos; (2) women with tattoos would perform worse than women without tattoos on the IGT; (3) there would be a significant correlation between the BIS-11score and the IGT performance among women with tattoos, and (4) Motor, non-planning and attentional impulsiveness factors of the BIS-11 as expressions of specific facets of impulsivity would be related to specific neuropsychological mechanisms of risky decision-making in young tattooed women (5) smoking status, however, would not explain these associations in the tattooed women.

## Methods

All participants (tattooed women and control women) were recruited by means of advertisements posted at universities, through personal contacts and via social networks (Facebook), to take part in a research project investigating decision making styles between tattooed women and those without tattoos. All participants were recruited throughout the Tel Aviv area between March 2012 and July 2012. The participants in both groups (research and control) were either employed or students or graduates and belonged to similar socioeconomic backgrounds. The participation in the study was voluntary and without payment. As compensation for participating in the study participants received free charge consultation regarding their decision-making style as well as professional advice regarding the results of the neuro-cognitive and personality assessments. Individual sessions were conducted for the purpose of the study. Participants were given an explanation regarding the research aims and signed a consent form indicating their willingness to participate in the research which included the computerized neuropsychological examination, the personality tests, and a special questionnaire covering extensive background information. The duration of each individual session was up to an hour and a half, with the entire research process taking place over a period of five months.

Sixty women volunteers with tattoos aged 18 to 35 years (M = 28.4, SD = 5.95) were included in the study. Fifty eight percents of the tattooed women had more than one tattoo. All the participants were tattooed at the time of the study (women with removed tattoos were not included). The participants were either employed or students with the following education level: high school diploma or lower – 46.7%, undergraduate university degree – 25%, Master’s university degree– 23.3%, or Doctoral degree – 5%.

We analyzed only tattooed women in order to avoid gender differences on the IGT performance [57]. We expected that comorbid neurological problems, alcohol use disorders and drug dependence would result in an additive effect on neuro-cognitive deficiencies [53]. Thus, the exclusion criteria were neurological disorders, mental retardation, alcohol and substance abuse/dependence (other than tobacco smoking), major psychiatric disorders and treatment with any psychiatric medication. It was established that 55% of participants from the tattooed group were smokers. A semi-structured interview of 20-items to measure the tattooed women’s characteristics was administered by one of the researchers (AK).

The control group included 60 healthy volunteers in a similar age range as the study group, namely 18–35 years old (M = 28.5, SD = 5.43) and recruited from the same area. Education level in the control group was as follows: high school diploma or lower – 25%, undergraduate university degree – 28.3%, Master’s degree – 41.7% and Doctoral degree – 5%. All participants completed a screening interview, which covered the following areas: medical history, illicit drug use, family and personal psychiatric history. None of the subjects received any psychopharmacological treatment. Exclusion criteria for the untattooed control women (C) included any current or past DSM-IV-TR axis I psychiatric disorder. Only 10% of participants from this group smoked regularly.

The study was approved by the Bar-Ilan University Ethics Review Board (Ramat Gan, Israel).

### Decision making measures: computerized animated variant of the Iowa Gambling Task (IGT)

We applied a modified computerized animated time-unlimited version of the IGT [58] - Casino task (AnimaScan Ltd, Ashdod, Israel, 2000), for details see [52]. Briefly, the IGT requires individuals to select cards from four different decks, called A, B, C, and D. Two of the decks, A and B, often result in high gains ($100) but also carry a high risk of large losses thus leading to a cumulative long-term loss. Therefore, these decks are disadvantageous (“bad”). The remaining two decks (labeled C & D) typically result in lower rewards ($50), but also generate lower losses, resulting in a cumulative long-term gain and are therefore advantageous (“better”).

Prior to starting the IGT, the participants were told that the goal of the game was to win as much money as possible and avoid losses to the best of their abilities. They were told that they may choose cards from any deck, and that they may switch decks at any time. Participants were also informed that some of the decks are more advantageous than others, and in order to win, one must avoid the “bad” decks and stick to the “better” ones [38], but they had to find out by themselves which decks were “bad” and which “better”.

Each participant chose 100 cards which were then analyzed by dividing them into 5 blocks of 20 cards each. We calculated a net score for each block by subtracting the number of advantageous cards from the number of disadvantageous ones [(C + D) - (A + B)] for each of the 20 card blocks. A score below 0 signified that subjects adopted an overall disadvantageous strategy (more card selections in decks A and B), while a score above 0 implied a more advantageous deck preference (more card selections in decks C and D).

### Barratt impulsiveness scale (BIS-11)

The Barratt Impulsiveness Scale (BIS-11) is a 30 item self-report instrument designed to assess the personality/behavioral construct of impulsiveness [59]. Participants rate the 30 statements on a 4-point Likert scale ranging from 1–4: 1 = never/rarely, 2 = sometimes, 3 = frequently and 4 = almost always/always. The higher the total score, the higher the self-reported level of impulsivity (total scores range from 30 to 120). The BIS-11 is the most commonly administered self-report measure for the assessment of impulsiveness in both research and clinical settings [60].

Factor analysis of the BIS-11 reveals three subscales: motor impulsiveness, which reflects action without forethought (i.e. I do things without thinking); non-planning impulsiveness, reflecting the focus being on the present (i.e. I am more interested in the present than the future); and attentional impulsiveness that reflects reduced ability to maintain attention on a stimulus (i.e., I do not 'pay attention’; See [59] for a list of items comprising BIS-11 subscales).

### Statistical procedure

The data was analyzed using the SAS v9.1 statistical software package for Windows (SAS Institute Inc., Cary, NC, USA). The first step examined the difference between groups on socio-demographic variables (age, education and smoking habit). *T*-test was used to analyze numerical variables (age and education) and a chi-square (*χ*^2^) test for the categorical variable (smoking habit). In the second step Pearson’s correlation test was used to assess the relationships between the demographic characteristics, self-report measures of the BIS-11 and the IGT performance.

In the third step MANCOVA was used to examine the influence of the group (tattooed or control) on common self-report measures of impulsiveness (BIS-11 subscales) while controlling for socio-demographic characteristics. To analyze the effect of group differences in the decision making process (the five blocks of the IGT), repeated measures ANCOVA was performed with socio-demographic characteristics as covariates. Since ANCOVA assumes that a dependent variable is linearly related to the covariates, correlation analysis was performed to evaluate the relationship between socio-demographic characteristics and behavioral measurements.

## Results

### Between-group comparison of the socio-demographic characteristics

Univariate analysis did not show differences between groups with regard to age (t = 0.11, df = 118, p = 0.91), but significant differences were found regarding education (t = 2.60, df = 118, p = 0.01). Women with tattoos were less educated. A significant difference was also found on smoking habit (*χ*^2^ = 27.69, df = 1, p < 0.0001). The proportion of smokers in the tattooed group was 5 time higher than in the control group (Table 1).

**Table 1:** Descriptive statistics of demographic characteristics, measures of the Barratt Impulsiveness Scale -11 and the Iowa Gambling Task performance in women with and without tattoos

	**Tattooed (n = 60)**		**Non-tatooed (n = 60)**	
	**Mean**	**SD**	**Mean**	**SD**
Age	28.47	5.42	28.35	5.95
Education	14.53	2.77	15.82	2.63
Smoking	55%	-	10%	-
**BIS-11**				
AI	16.91	3.66	14.42	2.98
MI	20.83	4.02	17.57	2.98
NPI	24.88	3.82	22.03	3.84
**IGT**				
Block 1 (1–20)	-1.2	3.54	-1.46	4.21
Block 2 (21–40)	-0.97	3.24	0.47	5.20
Block 3 (41–60)	0.6	5.74	1.3	7.07
Block 4 (61–80)	-0.5	5.85	2.37	8.25
Block 5 (81–100)	-0.17	7.24	4.53	7.82

*BIS-11* Barratt impulsivity questionnaire, *AI* attentional impulsiveness, *MI* motor impulsiveness, *NPI* non-planning impulsiveness, *IGT* Iowa Gambling Task as in Bechara et al. (1994), the 100 card selections from the IGT were sub-divided into five blocks: 1–20, 21–40, 41–60, 61–80, and 81–100.

Because education and smoking habit were found to be significantly different between groups they were considered as potential covariates. MANCOVA analysis assumes that the dependent variable should be linearly related to the covariate (education). Correlation analysis showed no linear relationship between impulsivity measures and education (Table 2). Since there was also no relationship between level of education and IGT scores, education was excluded from the list of potential covariates.

**Table 2:** Correlations between demographic characteristics, Barratt Impulsiveness Scale (BIS-11) measures and Iowa Gambling Task (IGT) performance

	**Age**	**Education**	**Smoking**	**BIS-T**	**AI**	**MI**	**NPI**	**Block 1 (1–20)**	**Block 2 (21–40)**	**Block 3 (41–60)**	**Block 4 (61–80)**	**Block 5 (81–100)**
Age	1		-0.1195									
Education	0.6587^***^	1	-0.3213^**^									
Smoking			1									
BIS-T	0.0289	-0.1266	0.3035^***^	1								
AI	-0.0387	-0.1051	0.1610	0.7471^***^	1							
MI	-0.0369	-0.2125^*^	0.3779^***^	0.8021^***^	0.4189^***^	1						
NPI	0.1330	0.0137	0.1716	0.7996^***^	0.3843^***^	0.4581^***^	1					
Block 1	-0.0899	-0.1266	-0.06412	0.2158^*^	0.192^*^	0.132	0.1848^*^	1				
Block 2	-0.1937	-0.1448	0.0398	-0.0971	-0.0541	-0.0653	-0.1058	0.0374	1			
Block 3	0.0520	0.1152	-0.0585	0.0462	0.0243	-0.0151	0.0956	0.1180	0.0623	1		
Block 4	-0.0138	0.0018	-0.0403	-0.0418	-0.1024	-0.0055	0.0019	-0.0304	0.0476	0.1371	1	
Block 5	0.0242	0.0093	-0.0932	-0.1749	-0.0986	-0.2041^*^	-0.1068	-0.0171	0.0988	0.0549	0.0832	1

*BIS-T* total BIS-11 score, *AI* attentional impulsiveness, *MI* motor impulsiveness; and *NPI* non-planning impulsiveness; The 100 card selections from the IGT were sub-divided into five blocks: 1–20, 21–40, 41–60, 61–80, and 81–100.

^*^p < .05; ^**^p < .01; ^***^p < .005.

### Between-group comparison of the IGT performance

**Figure 1:**
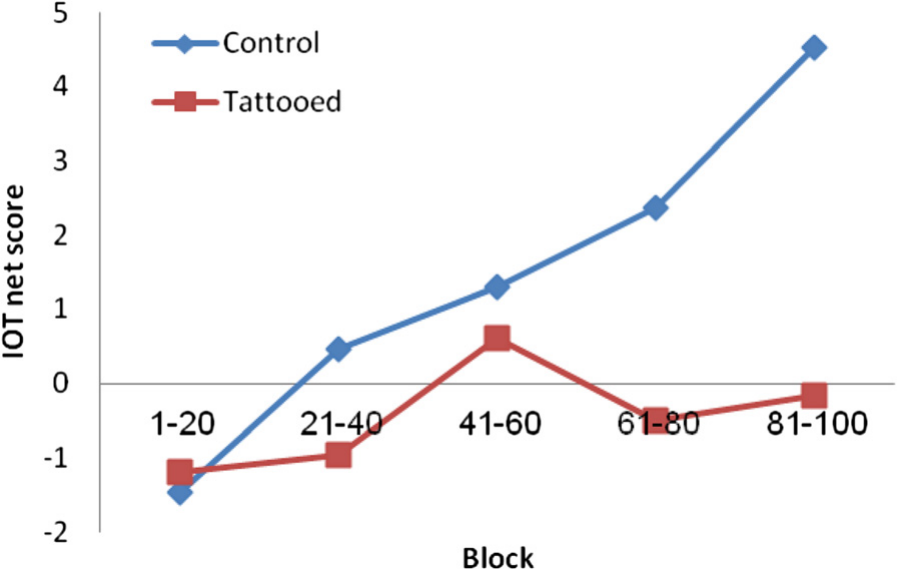
**Mean Iowa Gambling Task net scores on each of the five blocks.** The performance of the tattooed and non-tattooed groups. Performance in IGT block 1, block 2 and block 3 did not differ between the two groups, but the differences between group differences became significant in blocks 4 and 5. The positive net score of the non-tattooed group from block 2 to 5 can be explained by their fast learning in contrast to the tattooed women, who did not improve during the task and exhibited non-optimal outcomes because they failed to correct disadvantageous choices.

A repeated measure ANCOVA was used with the groups (tattooed vs. controls) as between-subjects factor, block (each 20 trials) as a within-subjects factor; smoking status as covariate and the net score of IGT as the dependent variable. The results showed an effect of block and an effect of “group x block” interaction: F(4,116) = 3.49, p = 0.0103, indicating that task performance increased consecutively from block to block; F(4,116) = 3.64, p = 0.0081, and that the women with tattoos performed worse than controls (see Figure 1) on the second, fourth and fifth block of IGT (Table 3). The control group improved significantly their learning curve. Such an improvement was not obtained in the tattooed women. There was no group effect after adjustment for smoking habit F(1,119) = 3.87, p = 0.051.

**Table 3:** Interaction between each group and Iowa Gambling Test’s blocks: pairwise comparisons of performance measures

**IGT**	**Adjusted mean**		**P**
	**Tattooed**	**Non-tattooed**	
Block 1 (1–20)	-0. 7394	-0. 9333	0.8148
Block 2 (21–40)	-1.2076	1.0167	0.0181^*^
Block 3 (41–60)	1.1424	2.3083	0.3956
Block 4 (61–80)	-0.6288	2.9417	0.0226^*^
Block 5 (81–100)	-0.2803	5.15	0.0012^**^

^*^p < .05; ^**^p < .01 Pairwise post-hoc comparisons were performed and corrected for multiplicity using the Tukey-Kramer test.

### Between-group comparison of the barratt BIS-11 measures

A one-way (group) MANCOVA revealed significant multivariate group effect (Wilk’s Lambda = 0.82, F(3,114) = 8.57, p < 0.0001) in all BIS-11 subscales: motor impulsiveness, non-planning impulsiveness, and attentional impulsiveness scores (Table 4), indicating that the groups differed significantly on the combined set of BIS-11 subscales.

**Table 4:** Analysis of Covariance (MANCOVA) on impulsivity measures

**BIS-11**	**F**	**P**	**Adjusted mean**	
			**Tattooed**	**Control**
**AI**				
Gr	13.27	0.0003	17.04	14.69
Smoking	0.15	0.7008		
Smoking x Gr	1.09	0.2980		
**MI**				
Gr	13.09	0.0004	20.55	18.38
Smoking	7.90	0.0058		
Smoking x Gr	1.89	0.1721		
**NI**				
Gr	16.01	0.0001	25.08	22.61
Smoking	0.76	0.3848		
Smoking x Gr	3.23	0.0748		

*BIS-11* Barratt Impulsiveness Scale, *AI* attentional impulsiveness, *MI* motor impulsiveness; and *NPI* non-planning impulsiveness, *Gr* study groups (tattooed vs. non-tattooed).

No overall smoking effect was found on BIS measures as shown by Wilk’s Lambda = 0.93, F(3,114) = 2.68, p = 0.05. Neither was an overall “smoking x group” interaction effect found, as shown by Wilk’s Lambda = 0.97, F(3,114) = 1.3, p = 0.28. As expected, examination of the BIS-11 subscales separately, using univariate test, showed that women with tattoos self-rated themselves significantly higher on all impulsivity domains than controls (Table 4). Smoking habit was significant only for Motor Impulsivity (F = 7.90, p = 0.0058). Interaction between smoking habit and group was not significant on all measures. The adjusted means of impulsivity measures were slightly larger than unadjusted means (Table 4).

### Association between risky decision-making and impulsiveness measures

Table 2 shows absence of association between self-assessed impulsivity measures on the BIS-11 scales and risky decision-making on the IGT, indicating that these two measures assess different aspects of risk taking behavior.

## Discussion

The purpose of this study was to determine if measures of impulsivity (the BIS-11) and risky decision-making (the IGT) are associated with tattoo-related behavior in a sample of young healthy women. Our hypothesis was that both self-reported impulsiveness (BIS-11) and risky decisions in IGT, can predict independently the tattoo-related behavior. Our results indicate that both impulsivity (as measured by a questionnaire) and risk-taking decisions (as measured by a behavioral task) predicted presence of tattoos.

### Risky decision-making among tattooed women

To the best of our knowledge this is the first experimental study of the decision making process in tattooed women. It was found that risky decision-making was associated with the selection of big gains albeit with maximal losses (the disadvantageous decks). In contrast, non-risky decision-making was associated with shifting selections to smaller gain but minimizing losses (the advantageous decks). The development of successful decision-making strategies after multiple evaluations of winning and losing are associated with higher net scores [58].

In the initial phase of the IGT the subjects make choices in conditions of maximal uncertainty. The decisions in the “ambiguity” phase were most likely made without awareness to the probabilities of reward or loss [61]. Decisions in the initial phase of the IGT did not differ between tattooed women and controls. This result is expected since random responses are a common strategy in uncertain situations [62].

The second part of the IGT performance constitutes “decision-making under risk” [47], in which subjects become more knowledgeable on the risks associated with each deck. The performance of the tattooed women differed significantly only at this stage: controls gradually shifted to advantageous decisions while the tattooed women continued to make disadvantageous decision as the task progressed (see Figure 1). Risky decision-making by tattooed women may result from their impaired ability to learn from the association between the actions and subsequent negative outcomes.

### Association between impulsivity and risky decision-making among tattooed women

The main hypothesis of the study was that tattooed women make risky decisions as a result of their impulsiveness. Trait impulsivity, as measured by BIS-11, is associated with learning impairment in problem solving situations [63]. The BIS-11 measures the tendency of individuals to consider negative consequences before acting [64]. Buelow and Suhr [39] found that riskier IGT performance is related to higher levels of sensation seeking and impulsivity. Risky performance in the IGT may result from a personality trait that causes participants to minimize their consideration of future consequences, either positive or negative [65]. Thus, the high self-reported impulsiveness on the BIS-11 scale is expected to reflect the risky decision-making on the IGT. The use of multifactorial impulsivity scales enables detection of specific facets of impulsivity, related to risky decision making (Table 2). This is the first study that uses a neurocognitive measurement of risky decision making and shows that, in contrast to our hypothesis, risky decision making in tattooed women is not a result of self-reported impulsiveness. According to Bechara [66] impulsiveness is fundamentally different from risky decision-making. The latter involves choosing wrongly when presented with several alternatives, while impulsivity represents inhibition dysregulation. A decision process requires weighing the pros and cons of various choices against each other and acting based on the results of this comparison. Although there is some overlap between trait impulsiveness and risky decision-making [67], it appears that they may represent separate independent entities. Our findings are consistent with previous studies indicating that the dimensions of impulsiveness are distinct and uncorrelated to risky decision-making [47-53].

### Association between tattoos and smoking in women

Similar to previous studies, we found a significant association between tattooing and smoking [15,54]. High impulsivity and risky decision-making in the tattoo group may be related to smoking as a confounding factor. Previous studies clearly implicate impulsivity as a precursor for smoking [see [68-71]]. In addition, association between tattooing and smoking may be related to the fact that both may have addictive characteristics [see for review: [72]]. Non-smoking tattooed women were more impulsive than non-smoking controls, as assessed by the BIS-11. In contrast, in smoking participants the between-group differences in the BIS-11 scores were lost. Thus it seems that impulsivity measures in tattooed women are smoking-independent. Namely, the smoking status is not a direct mediator of tattooing behavior.

### Limitations

Certain limitations impact the interpretation of our results. First, we did not examine comorbid Axis II disorders in the tattooed women, which may account for the neuropsychological findings. Future studies on this topic should assess personality psychopathology using appropriate structured clinical interviews. Second, this study was conducted among women with relatively small numbers of tattoos and without piercings who may be less impulsive than heavily tattooed or body pierced women [73,74]. Third, participants with alcohol and drug use comorbidity were excluded. The presence of both tattoos and substance use disorders may have exerted additive effects on the IGT performance in the disadvantageous direction.

## Conclusion

The present study was specifically designed to resolve questions regarding associations between impulsiveness and risky decision-making in tattooed women. It found that in this case, risky decisions are not a direct result of their self-reported impulsiveness.

Women with tattoos show substantial differences in decision-making from women without tattoos. Women with tattoos exhibited higher scores in the BIS-11, and failed in decision making tasks such as the IGT. But their impairments do not provide definitive evidence against a close association between impulsiveness and decision making. The association between impulsiveness and tattooing behavior was found to be independent of smoking status. There are a variety of environmental, social and psychological variables that lead to tattooing in young people. Our study highlights that impulsiveness and risky decision-making may be key factors in identifying individuals who are at risk for tattooing behavior. Identifying contributing factors could limit risky decisions associated with tattooing. The complex relationship among tattooing, impulsiveness, risky decision-making, Axis I and II psychopathology, gender, substance use disorders, sociodemographic charachteristics and particular brain mechanisms underlying the impaired decision making process merits large-scale investigation.

## Competing interests

SK is an employee of Anima Scan Ltd. Neither financial nor material support was received from any external resource for this work.

## Authors’ contributions

SK has built the design of the study and methods used for the study and the interpretation of results, drafted the manuscript and contributed to the final manuscript. AK recruited participants, conducted the interviews and contributed to the final manuscript. MV coded and analysed the data. Supervised the manuscript writing: RL and AW. All authors read and approved the final manuscript.

## Pre-publication history

The pre-publication history for this paper can be accessed here:

http://www.biomedcentral.com/1471-244X/13/278/prepub
